# Thermal characteristics of non-biological vessel phantoms for treatment of varicose veins using high-intensity focused ultrasound

**DOI:** 10.1371/journal.pone.0174922

**Published:** 2017-04-06

**Authors:** Mi-sun Kim, Ju-Young Kim, Si-Cheol Noh, Heung-Ho Choi

**Affiliations:** 1 Medical Device Evaluation Department, National Institute of Food and Drug Safety Evaluation, Cheongju, Chungcheongbuk-do, Korea; 2 Department of Biomedical Engineering, Inje University, Gimhae, Kyoungsangnam-do, Korea; 3 Department of Radiological Science, International University of Korea, Jinju, Kyoungsangnam-do, Korea; "INSERM", FRANCE

## Abstract

The ultrasonic treatment of varicose veins uses high-intensity focused ultrasound, in which a blood vessel is contracted by converting acoustic energy into thermal energy. In this study, we propose a phantom of varicose veins that can be applied for the efficient evaluation of ultrasonic treatment in varicose veins. The proposed phantom consisted of glycerol base tissue equivalent material, vessel mimic tube, and blood mimic substances. The vessel mimic tube was placed inner glycerol phantom and it was filled with blood mimic substances. Blood-mimicked substances are prepared by adjusting the concentration of the glycerol solution to be similar to the acoustic properties of the blood, and vessel-mimicking materials are selected by measuring acoustic properties and thermal shrinkage of various materials in a heat-shrinkable tube. The blood vessels surrounding the tissue are replaced with the phantom similar to glycerol-based organization, and venous blood flow is implemented using a DC motor. The heating characteristics according to the ultrasonic wave using the manufactured varicose veins phantom were evaluated. As the sound wave irradiation time and power increased, the contractility of the vessel mimicking materials and the temperature of the surrounding tissues were increased. When the blood-mimicking material was circulated, the highest temperature in the focused region and the contractility of vessel mimicking materials were reduced under the same conditions as used for sonication. The manufactured phantom may contribute to the treatment of varicose veins and can be used to predict the ultrasonic therapeutic efficiency of varicose veins.

## Introduction

Varicose veins is a blood reflux disease that superficial veins visibly protruding out of the skin. It cause high venous pressure and valvular dysfunction caused by protruded superficial veins. This condition is mainly observed in women who stand or sit in one place for long periods of time. Worldwide, varicose veins occur in 10–15% of males and 20–25% of females, indicating a relatively high prevalence [1]. Treatment is necessary because varicose veins can cause aesthetic problems and cardiovascular disease. Varicose vein treatments include vein surgery, sclerotherapy drugs, varicose radio frequency closure, and the intravenous laser therapy. These treatments can cause wound complications and neurological damage, hematoma and blood clots in veins, and phlebitis as side effects [2]. In order to reduce these side effects, a non-invasive varicose vein treatment is needed. Recently, the possibility of varicose vein treatment using high-intensity focused ultrasound (HIFU) has been proposed.

Ultrasonic treatment of varicose veins uses HIFU involves contraction of the blood vessel using thermal energy and does not require closure or elimination of blood vessels, reducing the side effects of conventional invasive treatment options. In the 2000s, because of the increasing social interest in varicose veins treatment, *in vitro* studies for non-invasive treatments were conducted using a phantom and laboratory animals [3, 4]. Salomir et al. performed temperature mapping using magnetic resonance imaging during HIFU to investigate fresh porcine tissue specimens. This study reported that a thermal increase by HIFU sonication could shrink the vein [5, 6]. Pichardo et al. evaluated vessel shrinkage by investigating 3 MHz HIFU, targeting one phantom inosculate human understated vein and porcine muscle tissue and phantom inosculate superficial veins extracted from human and porcine muscle tissue [7]. Henderson et al. confirmed the applicability of varicose vein treatment by 1.54 MHz HIFU using the inosculate phantom with rat skin, sheep muscle and vessel, and porcine fat tissue [8]. Although these studies produced *in vitro* phantom and not vessel mimic substances using actual laboratory animals and human veins, the exclusion of blood flow made it difficult to maintain the form of the blood vessel [9]. Additionally, the accuracy and reproducibility of the evaluation were decreased by biological alteration of the blood vessels over time through tissue and biomechanical changes. Ushijima et al studied the relationship between sonication conditions and skin burns by applying the HIFU to a bovine serum albumin phantom [10, 11]. As described above, studies on the sonication conditions for effective treatment with minimized skin burns are required. The development of tissue-mimicking varicose vein phantom should be examined.

In this study, we present a material with acoustic characteristics similar to the vascular tissue and blood. A tissue-mimicking varicose vein phantom enabling intravenous blood flow in patients and maintenance of blood vessel in the surrounding tissue is proposed. In order to evaluate the usability of the prepared phantom, we confirmed an increase in the temperature of the surrounding tissue and contractility of vessel mimicking materials. This was accomplished by using ultrasonic irradiation conditions at 1.1 MHz HIFU and compared with the shrinking of the actual vascular tissue. The tissue mimicked varicose vein phantom may contribute to the treatment and ultrasonic therapeutic efficiency of varicose veins.

## Related researches

**Fig 1:**
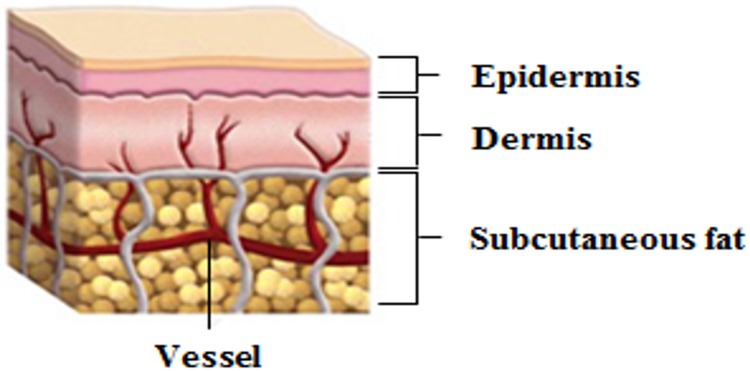
Veins and surrounding tissue.

Veins generally have large diameters and irregular, thin walls, and are less resilient than arteries. Veins contain valves that prevent the reverse flow of blood against gravity in the venous lumen. Varicose veins are divided into deep veins and shallow veins depending on the depth. These veins are distributed in the subcutaneous adipose tissue in the shallow vein and subcutaneous adipose tissue in the upper covered skin tissue composed of epidermis and dermis. The great saphenous vein causes branching of varicose veins located in a depth of 10–15 mm from the skin surface. Fig 1 shows the structure of a shallow vein and the surrounding tissue. In addition, varicose veins can be divided into telangiectasia, shape spider varicose veins, reticular varices, and branch varicose veins depending on vessel diameter. Table 1 shows the characteristics of typical veins and varicose veins.

**Table 1:** Characteristics of normal and varicose veins.

category		inner diameter	wall thickness
normal vessel	capillary	6.0 μm	1.0 μm
	venule	20 μm	2.0 μm
	vein	5.0 mm	0.5 mm
varicose veins vessel	capillary	0.1–0.2 mm	-
	venule	2.0–4.0 mm	-
	vein	4.0–8.0 mm and over	0.46–0.64 mm

Because the veins are less affected than the arteries by the heartbeat, the characteristics of blood flow are not significant, and little change occurs in the blood flow. The velocity of blood flow measured in the popliteal vein of varicose veins patients is 4.0–15 cm/s. This value is low compared to in a normal subject (20–35 cm/s).

Unlike blood flow phantom used for diagnostic ultrasound, blood flow phantom for therapeutic ultrasound is rare. Moreover, there are some limitation to assess the heating characteristics and therapy quantitatively by using that phantom. In order to confirm the hemostatic effect of HIFU treatment, J. Huang produced a blood mimic substances using glycerin aqueous solution and a blood flow phantom using an agarose-based wall-less phantom [12]. However, because absent of vessel wall and lack of mechanical strength, it is not suitable for effect evaluation. Greaby et al. evaluated blood coagulation treatment of HIFU using pulsatile blood flow phantom consisting of porcine carotid arteries and a blood, agarose-based gel, and circulation pump [9]. This phantom was designed to be similar to the blood flow velocity, pressure, and beat of a real person; however, because of the use of actual blood vessels, a formaldehyde chemical process is required to prevent the corruption of blood vessels. For these reason, changes in the acoustic characteristics and biomechanical properties of the blood vessels were observed. Dasgupta et al. assessed the impact of the blood flow of the large vessel on heating HIFU temperature by creating a hydrogel-based tissue mimicked phantom and blood flow phantom consisting of a glass tube [13]. However, this phantom is inappropriate for the application of therapeutic ultrasound of the blood vessels in the treatment through targeted tissue mimicking phantom of proximal vessel-mimicking material. Therefore, in this study, we constructed varicose vein phantom to overcome these limitations. The structural features and blood flow of varicose veins were considered, and proposed phantom was composed entirely of non-biological materials for easy storage and to maintain the consistency of characteristic changes and shapes. These properties are thought to be useful for confirming reproducibility for repeated experiments.

### Materials and methods

**Fig 2:**
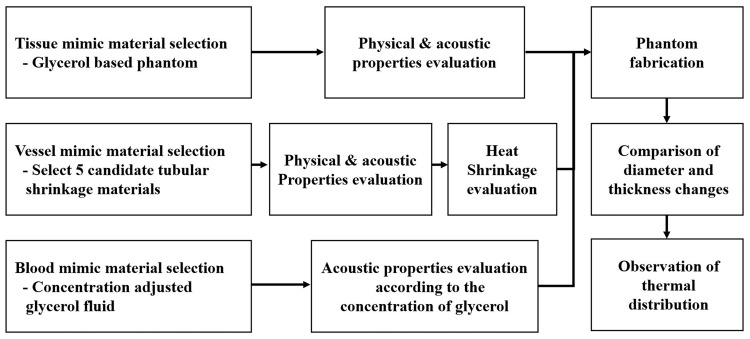
The flow-chart of this study.

In this study, we proposed the tissue equivalent phantom for treatment effect evaluation of ultrasound varicose vein. In addition, we confirmed the usefulness of proposed phantom by evaluating the physical and acoustic characteristics, and heat shrinkage. First, the glycerol-based tissue equivalents were fabricated and evaluated. Moreover, the suitable materials were selected by evaluating the candidate groups of the vessel mimic substances and blood mimic substances. Then the tissue mimic phantom was fabricated for the ultrasound varicose veins treatment using selected materials, and it was evaluated by observing the heat shrinkage and thermal characteristics. Finally, we compared the thermal properties of vessel mimic material and bovine vessel to confirm the usefulness of the phantoms presented in this study. The characteristics and performance evaluation of fabricated phantom were measured repeatedly (25 times for acoustic characteristic test and 5 times for heat-shrinkage and thermal characteristic evaluation). The measurement results were presented by using mean value and deviation. Fig 2 shows the flow-chart of this study.

### Fabrication of the varicose vein phantom

In this study, the phantom was produced with clinical characteristics similar to those of blood vessels and with the flow characteristics and surrounding tissue structure of actual patients. By adjusting the concentration of the glycerol solution, we prepared blood-mimicking material with acoustic properties similar to that of blood. By evaluating the various heat shrinkable tubular materials, materials with acoustic characteristic and heat shrinkage most similar to blood vessels were selected. Skin tissue and fat tissue were replaced with glycerol-based tissue mimicked material phantom, and blood-mimic material was allowed to flow with the varicose venous blood flow velocity in vessel mimic material using a DC motor (11.21± 1.86 cm/s). The concentration of the glycerol solution was adjusted to 0~40% in order to produce a material which has a similar acoustic properties of blood, presented in IEC 61685 standard. The vessel mimic material requires the similar acoustic properties and heat shrinkage characteristic with the blood vessel. For these reasons, among shrinkable tubes having elasticity and similar acoustic impedance, the characteristics of general rubber tube, silicon tube, polyolefin tube, and polyvinyl tube were analyzed and compared with the blood vessel. The inner diameter and thickness of each tube was in the range of 6~8.5 mm and 0.3~0.6 mm, which was similar to the branch varicose veins in the lower part of the veinlet. For conformity assessment of the non-biological material substance, comparative experiments were performed using bovine vascular tissue. The vein that was harvested from the bovine lungs slaughtered within an hour was used. Size of 4~13 mm blood vessel was used and it was storage in saline to remove the blood.

**Fig 3:**
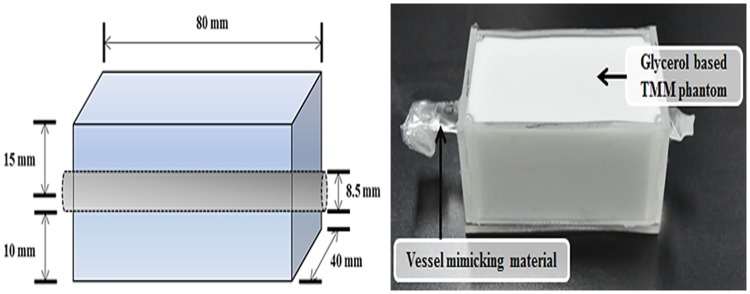
Structure and appearance of the fabricated phantom.

In order to replace the skin and fat tissue in the configuration tissue of varicose vein phantom, the glycerol-based tissue mimic phantom specified in IEC 60601-2-5 was used. The size of the phantom was made of 80 × 40 × 30 mm^3^, and the vessel mimic tube was located in a depth of 15 mm (location of great saphenous). Fig 3 shows the structure of the fabricated phantom.

### Acoustic characteristic and heat shrinkage evaluation

**Fig 4:**
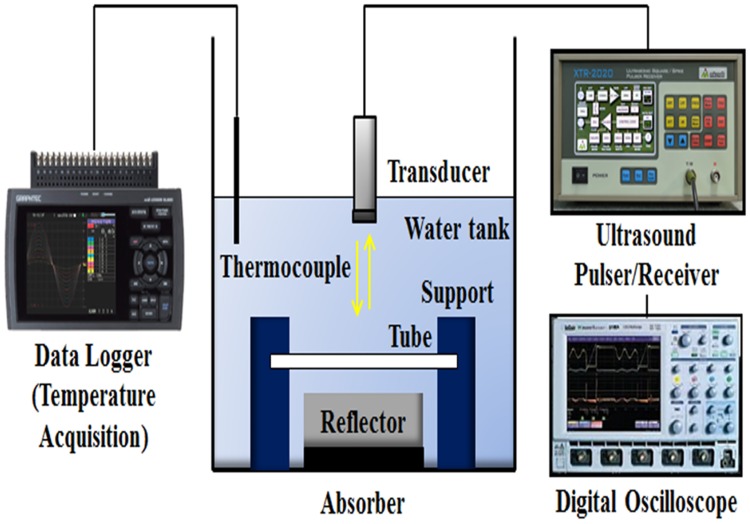
Experimental setup for acoustic characteristic evaluation of phantom.

In order to evaluate the blood-mimicking and vascular-mimicking materials used to prepare phantom varicose veins, we conducted acoustic characteristic evaluation by measuring each phantom and sound speed of vascular tissue and attenuation factor, density, and acoustic impedance. Fig 4 shows the experimental setup for measuring the acoustic characteristics of the phantom. After filling the tank with degassed water, the temperature was maintained at 37°C to have a similar environment to the body temperature, and ultrasonic wave transmission and reception were conducted using an ultrasound pulse/receiver (MKPR-1030, Korea Inc., Korea) and single transducer of 3.5 MHz (Aerotech Co., Pittsburgh, PA, USA). The received signal was stored using a digital oscilloscope (WaveRunner 6100A, LeCroy Corp., Santa Clara, CA, USA). Data were analyzed using Acknowledge 3.7.5 (BIOPAC Systems, Inc., Goleta, CA, USA).

**Fig 5:**
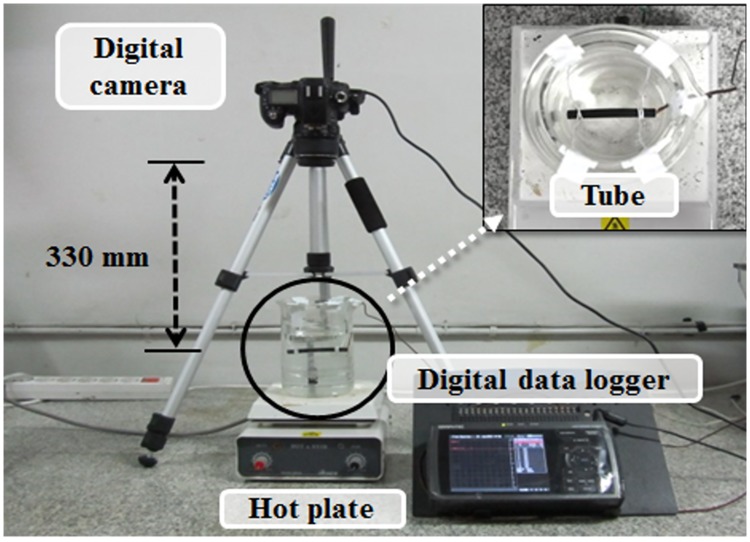
Experimental setup for evaluating heat-shrinkage of vascular-similar material and vascular tissue.

We compared heat-shrinkage for each tube and temperature increase in the blood vessel tissue for the heat-shrinkable vascular-similar material. The initial temperature of the sample was fixed at 25°C and heated in boiling water at 90°C using a hot plate. The temperature change was measured using a digital data-logger (midi logger GL820, Graphtech Co., Yokohama, Japan) and cross-sectional images of the sample were obtained using a digital camera (D90, Nikon Co., Tokyo, Japan) at increases of 1°C. The area of the sample was calculated using ImageJ 1.47v (National Institutes of Health, Bethesda, MD, USA) and MATLAB 8.0a (MathWorks, Natick, MA, USA). In addition, tube and heat-shrinkage of vascular tissue was calculated based on the sample area at an initial temperature of 25°C to compare the areas where the temperature increases. Fig 5 shows the experimental setup for investigating the heat-shrinkage of the vascular-similar material and vascular tissue.

### Evaluation of varicose vein phantom

**Fig 6:**
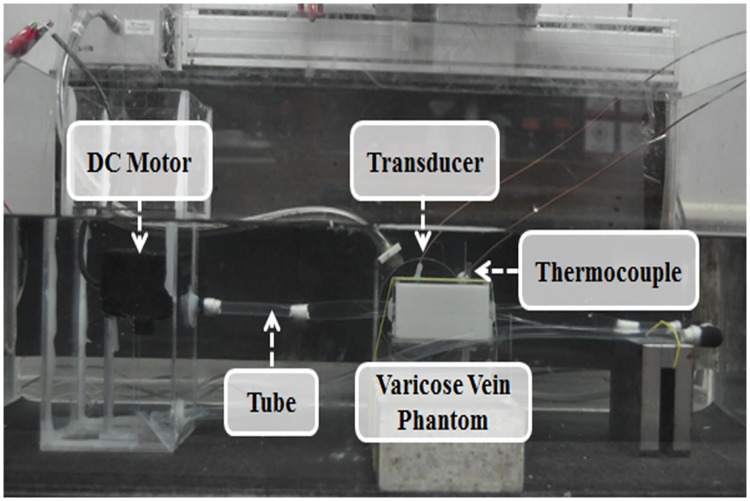
Experimental setup for shrinkage evaluation of the varicose veins phantom.

In order to measure the heat-shrinkage of the vascular-similar material and temperature of phantom, we observed the focused ultrasound surface of the vascular-similar material. We used a curved focused-transducer (H-101 model, SonicConcepts, Bothell, WA, USA) with a center frequency of 1.1 MHz applied as a burst wave (100 μs frequency, 3.5 Vp-p of 10 cycles) and continuous square wave. Electrical power in the power amplifier was set to 60–150 W at intervals of 30 W, and sonication time was set as three steps (60, 120, and 180 s). At this time, acoustic pressure on the sonicated power was 4.7, 6.24, 7.54, and 8.96 MPa and acoustic intensity was 737.6, 1298.8, 4620.4, and 2033.5 W/cm^2^. After examining the ultrasound, the B-mode images obtained before and after ultrasonic irrigation were obtained using ultrasound diagnostic imaging device and the contractility of vascular-similar material located in the varicose vein phantom was evaluated. The internal diameter of the vascular-similar material before and after shrinkage was measured as the distance function of the B-mode image. In addition, shrinking evaluation was conducted out considering the presence or absence of blood flow. Fig 6 shows the irradiated focused ultrasound experimental set-up.

In order to evaluate the temperature increase of varicose vein phantom according to the ultrasound irradiation conditions, a temperature sensor was used to evaluate 5 locations at 2-mm intervals. The distance from the surface of vascular-similar material was similar to that of the skin surface. After measuring the pulsed ultrasound of 30–150 W at intervals of 30 W for 180 s, we measured the temperature increase of vascular-similar material following irradiated power after cooling for 120 s. At this time, the time interval of data acquisition was set to 1 s, and the temperature was evaluated for each spot.

## Results

### Evaluation of characteristics of constituents

**Fig 7:**
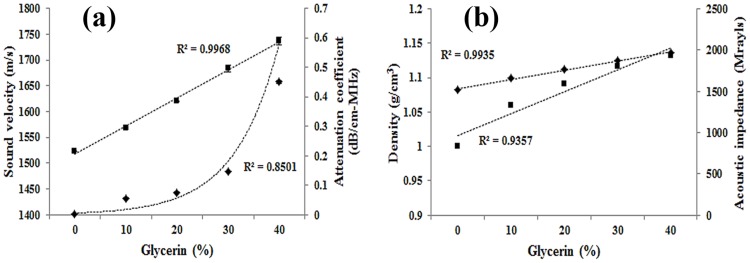
Acoustic characteristics trends according to concentration of glycerin solution; (a) sound velocity and attenuation, (b) density and impedance.

Fig 7 shows the graph of acoustic characteristics according to concentration of glycerin solution. Sound velocity, density and acoustic impedance increased linearly, and the attenuation coefficient increased exponentially as glycerin solution was increased from zero to 40%. According to the IEC 61685 standard, the acoustic characteristics of blood mimic material were as follows: 1570 ± 30 m/s of sound velocity, 0.1 dB/cm·MHz or less of attenuation coefficient, and 1050 ± 40 kg/m^3^ of density. Consequently, the 10% glycerin solution showing the most similar acoustic characteristics was determined to be the most suitable for use as a blood mimic material. Table 2 shows the acoustic properties according to the proportion of glycerin. The deviation of the acoustic characteristics according to the glycerin concentration is shown in S1 Table.

**Table 2:** Acoustic properties according to the proportion of glycerin.

Glycerin proportion	Sound velocity (m/s)	Attenuation coefficient (dB/cm·MHz)	Density (g/cm^3^)	Acoustic impedance (Mrayls)
0%	1,522.5±1.12	0.002±0.001	1.000±0.08	1.522±0.12
10%	1,567.6±1.69	0.056±0.001	1.060±0.07	1.662±0.11
20%	1,619.9±1.11	0.075±0.001	1.091±0.04	1.768±0.06
30%	1,683.3±4.90	0.145±0.002	1.117±0.03	1.879±0.05
40%	1,736.9±7.69	0.450±0.004	1.132±0.04	1.966±0.06

Table 3 shows the results of using four types of tubes and the measured acoustic characteristics for vascular-similar material. Bovine vascular tissue showed characteristics that were similar to the acoustic characteristics of human vascular tissue as reported in the literature (1513 ± 21 m/s of sound velocity, 1.45 dB/cm·MHz of attenuation coefficient, 1.066 g/cm^3^ of density, and 1.56 ± 0.03 Mrayls of acoustic impedance)[14]. The most similar material to the real vascular tissue in the four tubes experimented was the polyolefin shrinkable tube. Every characteristics of it except acoustic impedance was in error-range, and gap of acoustic impedance was under 0.1 Mrayls. The deviation of the acoustic characteristics of the vessel-mimicking materials and vessel tissues is shown in S2 Table.

**Table 3:** Acoustic properties of vessel-mimicking materials and vessel tissues.

Materials	Sound velocity (m/s)	Attenuation Coefficient (dB/cm·MHz)	Density (g/cm^3^)	Acoustic impedance (Mrayls)
General-purpose shrink tube	2,003.6±87.04	18.55±0.58	1.145±0.060	2.29±0.034
Silicone shrink tube	1,286.8±5.91	2.80±0.52	1.102±0.025	1.418±0.033
Polyolefin shrink tube	1,515.8±7.08	1.18±0.34	0.919±0.028	1.394±0.042
General- purpose vinyl tube	1,686.5±15.79	11.02±1.05	1.159±0.029	1.954±0.050
Bovine vessel tissue	1,510.0±68.99	1.45±0.418	1.108±0.060	1.670±0.08

**Fig 8:**
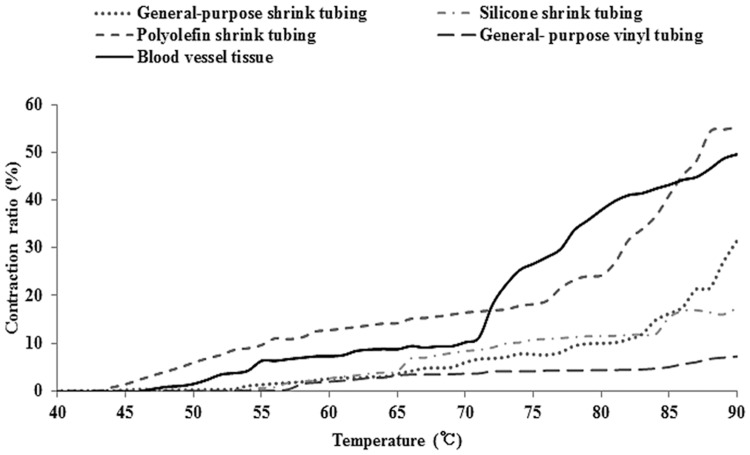
Heat shrinkage rate changes in the blood vessel mimic substances and vascular tissue according to the temperature.

Fig 8 shows the change in the thermal shrinkage ratio and vascular tissue with increasing temperature. The starting temperatures (47–51°C) of the silicone shrink tube and universal plastic tube were 53 and 54°C, respectively, confirming the difference between increasing the temperature interval of maximum heat-shrinkage and of rapid shrinkage. In contrast, the polyolefin shrinkable tube showed characteristics most similar to those of vascular tissue for heating characteristics, except for shrinkage starting temperature. By comparing the vascular tissue and acoustic characteristics of vascular similar material and heat shrinkage, the polyolefin shrinkable tube was suitable for use as a vascular-similar material.

### Evaluation of heat shrinkage of varicose vein phantom

**Fig 9:**
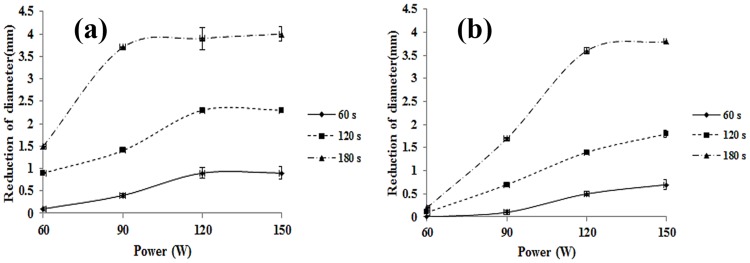
Diameter change of blood vessel mimic substances according to ultrasound exposure conditions; (a) non-BMF flow, (b) BMF flow.

**Fig 10:**
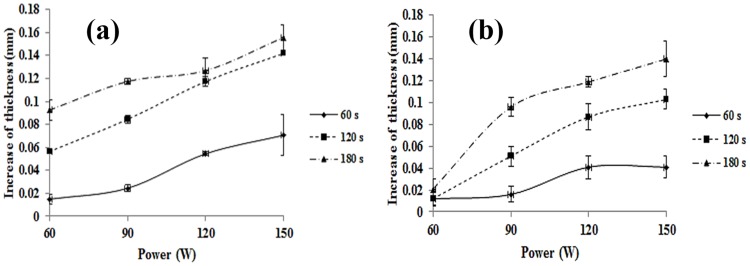
Thickness change of blood vessel mimic substance according to ultrasound exposure conditions; (a) non-BMF flow, (b) BMF flow.

After examining the effect of focused ultrasound with varying power and irradiation times considering or not considering the flow of vascular similar material, heat shrinkage was compared inside varicose vein phantom. Figs 9 and 10 show the change in the diameter of the vascular-similar material according to conditions of ultrasound irradiation. With increasing ultrasonic power and time at 60–150 W (30 W interval) and 60, 120, and 180 seconds, the inner diameter of vascular-similar material was reduced regardless of the flow of blood mimic material. However, at 180 seconds, the change (0.2 mm at 60 W, 3.8 mm at 150 W) in the maximum inner diameter was decreased compared with the non-recurring varicose vein phantom. Additionally, at 60 W for 60 s, the inner diameter was not reduced. In addition, the reduction of the inner diameter was very low. The circulation of blood mimic material was determined to have a greater effect on the shrinkage of vascular-similar material at lower acoustic power. This trend was observed under conditions that saturate reduced value of inner diameter. At 180 seconds sonication with BFM flow, the saturation of contraction of inner diameter was started at 90 W. By contrast, in the BMF flow condition, the saturation of contraction of inner diameter was started at 120 W.

This study used a blood vessel mimic substance with an internal diameter of 8.5 mm. For the valve of a blood vessel to perform as normal, the internal diameter of the vein must be decreased to 5 mm. This decreased diameter apply internal diameter contraction exposed 90 W power during 180 s in acyclic mode varicose vein condition. However, in cyclic mode, varicose vein conditions were 120 W power for 180 s, it is increased rather than acyclic mode varicose vein phantom.

**Fig 11:**
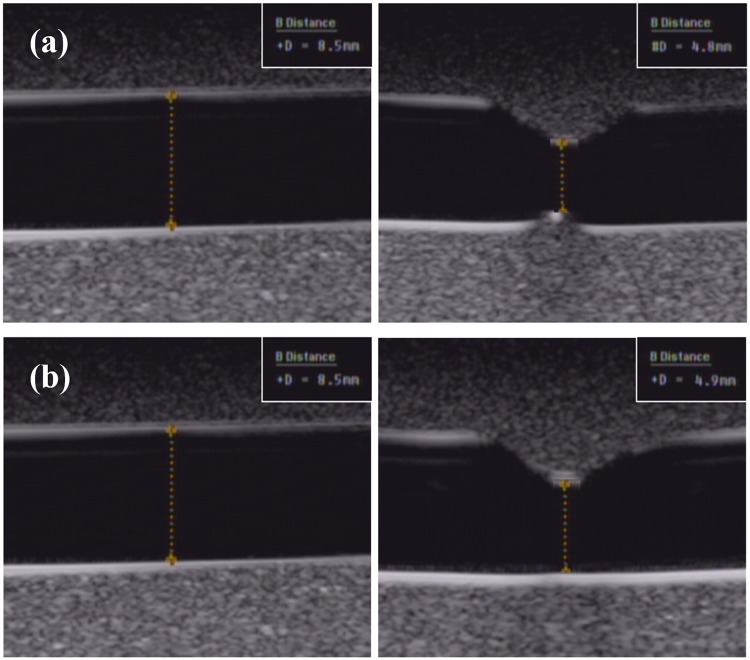
Ultrasound B-mode images of contraction in vessel mimic substance; (a) non-BMF flow, (b) BMF flow.

**Fig 12:**
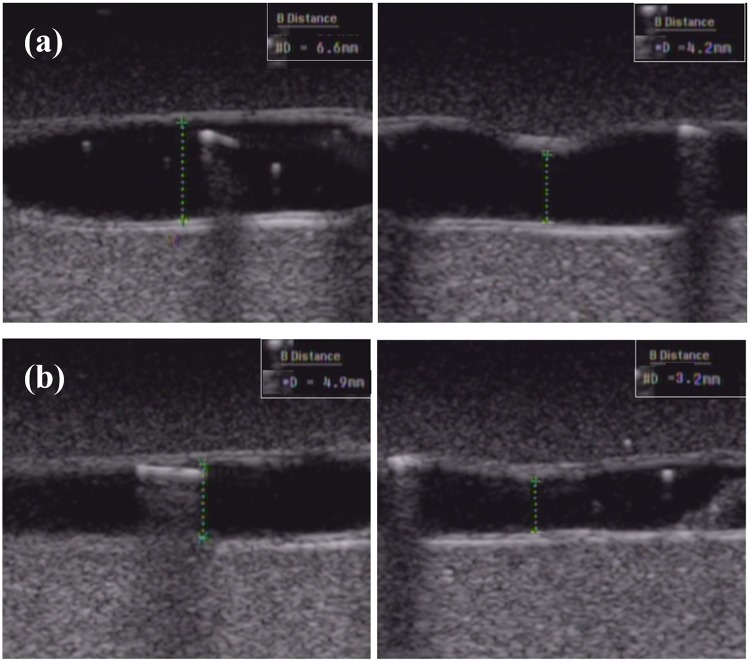
Ultrasound B-mode images of contraction in bovine vessel; (a) non-BMF flow, (b) BMF flow.

Figs 11 and 12 show the ultrasound B-mode images of contraction in vessel mimic substance and in bovine vessel under the non-BFM and BFM conditions. The diameters were decreased to 5 mm. If the blood mimic substance was not considered, the contraction percentage of the internal diameter was 35.71%. The blood vessel mimic substance showed contraction of 7.66%. The blood vessel internal contraction percentage in the cyclic mode was 35.59%. Varicose vein used with the blood vessel mimic substance was differed by 6.7%. The inner diameter contraction percentage of the blood vessel tissue in the cyclic mode was 35.59%, and the blood vessel mimic substance result of the varicose vein phantom differed by 6.7%. These differences in contraction blood of vessel tissue and blood vessel mimic substance showed similar results in terms of both the cyclic conditions and the acyclic conditions when heated in a water bath. However, the difference between the maximum contractions observed when considering the differences of 6% were considered to have been caused by differences in blood vessel tissue and blood vessel mimic substance with unique thermal contraction characteristics. Table 4 shows the change in diameter and thickness in vessel-mimicking material and vessel tissue with non-BMF flow and BMF flow.

**Table 4:** Change in diameter and thickness in vessel-mimicking material and vessel tissue with non-BMF flow and BMF flow.

		non-BMF flow		BMF flow	
		vessel-mimicking material	vessel tissue	vessel-mimicking material	vessel tissue
**Diameter (mm, %)**	**before sonication**	8.500 ± 0.010	6.66 ± 0.07	8.50 ± 0.01	4.94 ± 0.08
	**after sonication**	4.813 ±0.003	4.28 ± 0.06	4.91 ±0.67	3.18 ± 0.04
	**contraction rate**	43.376 ± 2.25	35.71 ± 2.10	42.29 ± 2.47	35.59 ± 0.97
**Thickness (mm, %)**	**before sonication**	0.299 ± 0.006	0.544 ± 0.005	0.299 ± 0.006	0.316 ± 0.030
	**after sonication**	0.416 ± 0.013	0.754 ± 0.043	0.418 ± 0.005	0.441 ± 0.056
	**increase rate**	39.3 ± 5.24	38.38 ± 7.19	39.8 ± 7.09	37.80 ± 4.23

### Evaluation of thermal characteristics of varicose vein phantom

The cyclic conditions of varicose vein phantom, including the maximum temperature and minimum temperature of the phantom at 60–150 W, was measured in channels 1 and 5, which was the same as the heating characteristics for the acyclic mode varicose vein. The maximum temperature measured in channel 1 was 82°C at 60 W, 88.1°C at 90 W, 90.6°C at 120 W, and 90.7°C at 150 W. When the power was increased, the maximum temperature was increased, showing a temperature difference of approximately 3–7°C when the cyclic condition and acyclic condition of the blood mimic substance were compared. This temperature decrease was observed in all channels; particularly, the temperature difference between channels 2 and 3 was 10–20°C. This is expected to lower the heat transfer to the surface around the focal point from the cooling effect of the circulation of the blood mimic substance.

In addition, when compared to the temperature of channel 5 corresponding to the tissue, except for the power of 150 W at 60–120 W, temperature did not cause tissue necrosis, and it is expected that burning of the skin surface would not occur under the conditions tested. As the result of measuring the temperature of the four channels corresponding to fat tissue, a temperature lower than 58°C was recorded only at 60 W of channel 4, and at 90 W or more of power, the threshold value above the temperature for the generation of local tissue destruction was observed in all channels. Accordingly, the fatty tissue of the skin proximal upon exposure with 60 W will likely not cause thermal damage; power levels greater than 90 W are expected to result in degeneration at all fat levels. The temperature measurement of acyclic mode varicose phantom showed that degeneration of the fatty tissue was unavoidable at all powers. Only at 90 W or less, skin tissue destruction is not expected to occur and the intensity increase of the threshold value can cause degeneration of the surrounding tissue by the circulation of the blood mimic substances. Table 5 compares the temperature measured at each channel in accordance with whether the blood mimic substance was circulating.

**Table 5:** Thermal increment rise in varicose vein phantom according to sonication conditions.

power (W)	circulation	channel				
		1	2	3	4	5
60	non-flow	90.5	90.3	83.6	65.4	40.8
	flow	82	70.3	63.5	57.9	40.7
	ΔT	8.5	20	20.1	7.5	0.1
90	non-flow	92.2	90.3	83.7	67.1	41.5
	flow	88.1	78	69.9	59.9	41.3
	ΔT	4.1	12.3	13.8	7.2	0.2
120	non-flow	92.7	90.3	85.6	71.1	47.6
	flow	90.6	79.3	72.8	60.2	41.8
	ΔT	2.1	11	12.8	10.9	5.8
150	non-flow	92.7	91	86.3	75.9	51.3
	flow	90.7	81.4	71.4	60.5	42.8
	ΔT	2	9.6	14.9	15.4	8.5

ΔT: Differences of the temperature between non-BMF flow and BMF flow

## Discussion and conclusion

In this study, we made a varicose vein phantom by using blood vessel and blood mimic substance and soft tissue mimic phantom replacing a blood vessel surrounding tissues. A DC motor was used to implement blood flow in the varicose veins in a patient, and in order to evaluate the usefulness of the proposed phantom, the contraction of the vessel mimic tube and the temperature rise in the surrounding tissue were observed. In addition we compared the contraction of blood vessel tissue and varicose veins tissue phantom using bovine vascular tissue. Blood vessel mimic substances used as varicose vein phantom have to similar heat contraction and acoustic characteristics with human tissue. For these reasons, in this study, to evaluate the suitability of proposed substance, we used a vein located in a bovine lung tissue to compare with the acoustic characteristics and heat contraction of the blood vessel mimic material. Actual varicose veins using a blood vessel of the patient in the study were evaluated for contraction according to HIFU exposure, and the contraction starting temperature and an increasing temperature of contraction percentage were 50°C and 70°C. A maximum contraction ratio of 59.5% at a temperature more than 81°C was observed. Compared with the heat contraction ratio, the contraction starting temperature and increasing temperature of the bovine pulmonary used in this study were very similar at 47–51°C and 70°C, showing a difference of 10% in the maximum contraction rate. This difference was related to the elasticity of blood vessels and pulmonary varicose veins, with the polyolefin contraction tube showing contraction up to 55.36%. This was confirmed to be more similar to the maximum contraction ratio of the actual varicose vein vessels. The difference in contraction ratio was observed when we considered the difference in bovine blood vessel tissue and the blood vessel mimic substance observed in heat contraction evaluation tests. The difference in the contraction ratio of the prepared phantom and blood vessel tissue of actual varicose vein patients were caused by differences in the thermal contraction characteristics. These conditions should be considered when the materials are used in humans. The acoustic property evaluation results of the actual bovine veins and blood vessel mimic substance, the damping coefficient, and sonic and the density characteristics of the polyolefin contraction tube were similar to those of vascular tissue. The difference in acoustic impedance did not show a difference of more than 0.1 Mrayls.

For the blood vessel mimic substance, the temperatures at which contraction began for the silicon contraction tube and a general-purpose plastic tube were 53 and 54°C, which were similar to the starting temperature of vascular tissue at 47–51°C; it was confirmed to be large difference at the maximum heat contraction ratio and rapid contraction ratio-increasing period. In contrast, polyolefin contraction tube showed the most similar characteristics with the blood vessel mimic substance except for the contraction starting temperature. Based on the vascular tissue and the acoustic characteristics of the blood vessel mimic substance and the heat characteristics, the polyolefin contraction tube was mostly suitable for use as a blood vessel mimic substance. Compare with the vascular tissue, these differences is smaller than previous study. Furthermore, due to the transparency proposed material has benefit of facilitate observation of flow during blood vessel contraction. Although the contraction starting temperature was 43°C, showing a difference between the contraction start temperature and the blood vessel tissue, the comparison of heat contraction and vascular tissue contraction ratio under these conditions were expected to be predictable.

Adjusting the concentration of the glycerol solution and evaluation of the acoustic characteristics showed that a glycerol solution of 10% had the most similar acoustic properties to the blood mimic substances specified in the IEC standard. In the flow phantom used to calibrate the diagnostic ultrasound equipment, it uses the blood mimic substance mixed various scattering body for evaluating the Doppler image. But because we aimed the evaluation of the applicability for treating varicose vein using ultrasound, we excluded the scattering body of the blood mimic substance, and we did not evaluate back scattering. In future study, if it is for varicose phantom to be used scattering body mixed with the glycerol solution of 10% and the scattering body of the same density, ultrasonic varicose veins in the treatment effect evaluation is determined to be able to apply with the Doppler device.

In this study, we discussed for phantom similar to human body in terms of acoustic characteristics, heat shrinkage degree and structure. There is no commercially available ultrasound varicose vein treatment device, so research for people is limited. For these reasons, although there is a difference from clinical environment, we conducted the evaluation using the bovine vessel, which has similar characteristics with human vessels. Structural and material limitations of the varicose veins treated using the phantom produced in this study may cause differences in treatment results when applied in the clinic. Blood flow affected the contraction and the vessel with increasing temperature inside the phantom, static vein *ex vivo* to the premise the blood flow block model varicose veins when compared to it, ultrasound in the treatment is expected to be more accurate and show better treatment outcomes. In addition to grafting skin tissue mimic phantoms, blood vessel mimic substances similar to the actual vascular structure, as well as the branch varicose veins, may be applied for the treatment of varicose veins and contribute to the treatment of varicose veins using ultrasound.

## Supporting information

S1 Table Acoustic properties according to the proportion of glycerin. (DOCX)

S2 Table Acoustic properties of vessel-mimicking materials and vessel tissues. (DOCX)
