# Supplementary material for: Thermal characteristics of non-biological vessel phantoms for treatment of varicose veins using high-intensity focused ultrasound
Source: PLoS One. 2017 Apr 6;12(4):e0174922. doi: 10.1371/journal.pone.0174922 (PMC5383065; doi:10.1371/journal.pone.0174922)
Supplement: S1 Table — (DOCX) [file pone.0174922.s001.docx]

**S1 Table. Acoustic properties according to the proportion of glycerin.**

|  | sound velocity (m/s) | | | | | Attenuation coefficient (dB/cm-MHz) | | | | | |
| --- | --- | --- | --- | --- | --- | --- | --- | --- | --- | --- | --- |
|  | 0% | 10% | 20% | 30% | 40% | 0% | 10% | 20% | 30% | 40% |  |
| 1 | 1522.7 | 1568.2 | 1620.8 | 1688.3 | 1728.5 | 0.002 | 0.056 | 0.077 | 0.146 | 0.449 |  |
| 2 | 1523.3 | 1569.4 | 1618.2 | 1683.7 | 1743.3 | 0.001 | 0.055 | 0.075 | 0.141 | 0.454 |  |
| 3 | 1522.6 | 1568.7 | 1620.3 | 1679.8 | 1737.2 | 0.003 | 0.054 | 0.073 | 0.142 | 0.453 |  |
| 4 | 1519.1 | 1566.7 | 1621.2 | 1684.8 | 1736.5 | 0.003 | 0.057 | 0.076 | 0.144 | 0.445 |  |
| 5 | 1523.2 | 1565.8 | 1619.7 | 1686.9 | 1734.8 | 0.001 | 0.055 | 0.075 | 0.149 | 0.451 |  |
| 6 | 1521.0 | 1567.2 | 1619.4 | 1688.4 | 1742.5 | 0.003 | 0.056 | 0.076 | 0.143 | 0.446 |  |
| 7 | 1522.8 | 1567.8 | 1619.9 | 1675.5 | 1736.5 | 0.001 | 0.056 | 0.077 | 0.144 | 0.442 |  |
| 8 | 1521.5 | 1567.2 | 1619.0 | 1679.9 | 1736.7 | 0.002 | 0.057 | 0.074 | 0.145 | 0.445 |  |
| 9 | 1523.6 | 1566.7 | 1620.4 | 1684.2 | 1730.5 | 0.002 | 0.056 | 0.075 | 0.146 | 0.449 |  |
| 10 | 1522.9 | 1569.3 | 1616.8 | 1671.8 | 1731.5 | 0.003 | 0.057 | 0.076 | 0.147 | 0.451 |  |
| 11 | 1523.2 | 1567.2 | 1619.3 | 1688.2 | 1742.8 | 0.001 | 0.055 | 0.077 | 0.145 | 0.453 |  |
| 12 | 1524.1 | 1567.9 | 1620.2 | 1684.9 | 1739.8 | 0.001 | 0.056 | 0.075 | 0.149 | 0.445 |  |
| 13 | 1521.9 | 1566.7 | 1621.8 | 1679.4 | 1720.5 | 0.003 | 0.059 | 0.073 | 0.144 | 0.454 |  |
| 14 | 1523.2 | 1569.2 | 1619.6 | 1678.6 | 1736.9 | 0.002 | 0.055 | 0.072 | 0.146 | 0.440 |  |
| 15 | 1523.8 | 1567.4 | 1619.2 | 1680.6 | 1752.5 | 0.002 | 0.054 | 0.076 | 0.144 | 0.451 |  |
| 16 | 1522.1 | 1568.4 | 1621.7 | 1683.4 | 1736.4 | 0.002 | 0.057 | 0.075 | 0.143 | 0.454 |  |
| 17 | 1522.9 | 1568.2 | 1619.9 | 1673.9 | 1728.5 | 0.003 | 0.054 | 0.074 | 0.141 | 0.451 |  |
| 18 | 1520.4 | 1569.6 | 1620.3 | 1687.2 | 1734.9 | 0.002 | 0.059 | 0.075 | 0.146 | 0.453 |  |
| 19 | 1522.9 | 1567.6 | 1618.1 | 1684.1 | 1735.6 | 0.001 | 0.056 | 0.075 | 0.144 | 0.456 |  |
| 20 | 1522.4 | 1564.3 | 1619.6 | 1684.3 | 1723.3 | 0.003 | 0.055 | 0.074 | 0.146 | 0.447 |  |
| 21 | 1522.7 | 1571.2 | 1620.9 | 1683.2 | 1736.8 | 0.003 | 0.057 | 0.076 | 0.145 | 0.451 |  |
| 22 | 1521.6 | 1563.6 | 1620.2 | 1685.8 | 1755.2 | 0.001 | 0.054 | 0.074 | 0.146 | 0.452 |  |
| 23 | 1522.2 | 1567.2 | 1620.6 | 1683.8 | 1740.9 | 0.001 | 0.057 | 0.075 | 0.140 | 0.450 |  |
| 24 | 1523.6 | 1568.2 | 1619.3 | 1692.4 | 1739.5 | 0.002 | 0.056 | 0.076 | 0.146 | 0.444 |  |
| 25 | 1522.7 | 1565.1 | 1619.9 | 1688.5 | 1742.1 | 0.002 | 0.055 | 0.075 | 0.143 | 0.452 |  |
| Average | 1522.5 | 1567.6 | 1619.9 | 1683.3 | 1736.9 | 0.002 | 0.056 | 0.075 | 0.145 | 0.450 |  |
| Standard deviation | 1.12 | 1.69 | 1.11 | 4.90 | 7.69 | 0.001 | 0.001 | 0.001 | 0.002 | 0.004 |  |

|  | Density (g/cm^3^) | | | | | Acoustic impedance (Mrayls) | | | | | |
| --- | --- | --- | --- | --- | --- | --- | --- | --- | --- | --- | --- |
|  | 0% | 10% | 20% | 30% | 40% | 0% | 10% | 20% | 30% | 40% |  |
| 1 | 0.8643 | 1.0662 | 1.1093 | 1.1159 | 1.1386 | 1.316 | 1.672 | 1.798 | 1.884 | 1.968 |  |
| 2 | 0.9138 | 1.0329 | 1.1111 | 1.0625 | 1.1438 | 1.392 | 1.621 | 1.798 | 1.789 | 1.994 |  |
| 3 | 0.9989 | 0.8415 | 1.0307 | 1.1561 | 1.1507 | 1.521 | 1.320 | 1.670 | 1.942 | 1.999 |  |
| 4 | 0.9789 | 1.1400 | 1.0912 | 1.1598 | 1.1310 | 1.487 | 1.786 | 1.769 | 1.954 | 1.964 |  |
| 5 | 1.0662 | 1.1010 | 1.0971 | 1.0813 | 1.1511 | 1.624 | 1.724 | 1.777 | 1.824 | 1.997 |  |
| 6 | 1.0579 | 1.1160 | 1.0553 | 1.0477 | 1.1317 | 1.609 | 1.749 | 1.709 | 1.769 | 1.972 |  |
| 7 | 1.0231 | 1.1277 | 1.0877 | 1.1829 | 1.1299 | 1.558 | 1.768 | 1.762 | 1.982 | 1.962 |  |
| 8 | 1.0463 | 1.0624 | 1.1235 | 1.0995 | 1.1482 | 1.592 | 1.665 | 1.819 | 1.847 | 1.994 |  |
| 9 | 0.9727 | 1.0672 | 1.1102 | 1.1145 | 1.1546 | 1.482 | 1.672 | 1.799 | 1.877 | 1.998 |  |
| 10 | 0.9383 | 1.0310 | 1.0935 | 1.1078 | 1.1366 | 1.429 | 1.618 | 1.768 | 1.852 | 1.968 |  |
| 11 | 1.0491 | 1.0835 | 1.0511 | 1.1172 | 1.0913 | 1.598 | 1.698 | 1.702 | 1.886 | 1.902 |  |
| 12 | 0.8681 | 0.8853 | 1.0900 | 1.1093 | 1.1317 | 1.323 | 1.388 | 1.766 | 1.869 | 1.969 |  |
| 13 | 1.1223 | 1.1157 | 1.1105 | 1.1236 | 1.1590 | 1.708 | 1.748 | 1.801 | 1.887 | 1.994 |  |
| 14 | 0.9992 | 1.1235 | 1.0669 | 1.0932 | 1.2321 | 1.522 | 1.763 | 1.728 | 1.835 | 2.140 |  |
| 15 | 0.9371 | 1.0265 | 1.0987 | 1.1121 | 1.1384 | 1.428 | 1.609 | 1.779 | 1.869 | 1.995 |  |
| 16 | 1.0131 | 1.1311 | 1.1864 | 1.1108 | 1.1501 | 1.542 | 1.774 | 1.924 | 1.870 | 1.997 |  |
| 17 | 0.8609 | 1.0675 | 1.0995 | 1.1255 | 1.1403 | 1.311 | 1.674 | 1.781 | 1.884 | 1.971 |  |
| 18 | 1.0681 | 1.0589 | 1.1121 | 1.1208 | 1.0761 | 1.624 | 1.662 | 1.802 | 1.891 | 1.867 |  |
| 19 | 1.0959 | 1.0258 | 1.1112 | 1.1710 | 1.1604 | 1.669 | 1.608 | 1.798 | 1.972 | 2.014 |  |
| 20 | 1.1114 | 1.1136 | 1.1250 | 1.0913 | 1.1188 | 1.692 | 1.742 | 1.822 | 1.838 | 1.928 |  |
| 21 | 1.0009 | 1.0648 | 1.0790 | 1.1431 | 1.0352 | 1.524 | 1.673 | 1.749 | 1.924 | 1.798 |  |
| 22 | 1.1067 | 1.0220 | 1.0610 | 1.1099 | 1.1372 | 1.684 | 1.598 | 1.719 | 1.871 | 1.996 |  |
| 23 | 0.9999 | 1.1007 | 0.9941 | 1.1278 | 1.1299 | 1.522 | 1.725 | 1.611 | 1.899 | 1.967 |  |
| 24 | 0.9084 | 1.1121 | 1.1104 | 1.1103 | 1.1003 | 1.384 | 1.744 | 1.798 | 1.879 | 1.914 |  |
| 25 | 0.9910 | 0.9840 | 1.0754 | 1.1193 | 1.0872 | 1.509 | 1.540 | 1.742 | 1.890 | 1.894 |  |
| Average | 1.000 | 1.060 | 1.091 | 1.117 | 1.132 | 1.522 | 1.662 | 1.768 | 1.879 | 1.966 |  |
| Standard deviation | 0.08 | 0.07 | 0.04 | 0.03 | 0.04 | 0.12 | 0.11 | 0.06 | 0.05 | 0.06 |  |
