# Supplementary material for: Thermal characteristics of non-biological vessel phantoms for treatment of varicose veins using high-intensity focused ultrasound
Source: PLoS One. 2017 Apr 6;12(4):e0174922. doi: 10.1371/journal.pone.0174922 (PMC5383065; doi:10.1371/journal.pone.0174922)
Supplement: S2 Table — (DOCX) [file pone.0174922.s002.docx]

**S2 Table. Acoustic properties of vessel-mimicking materials and vessel tissues.**

|  | sound velocity (m/s) | | | | |
| --- | --- | --- | --- | --- | --- |
|  | General-purpose  shrink tube | Silicone shrink  tube | Polyolefin shrink  tube | General- purpose  vinyl tube | Bovine vessel  tissue |
| 1 | 1914.6 | 1288.1 | 1510.2 | 1720.6 | 1427.1 |
| 2 | 1912.5 | 1286.1 | 1514.9 | 1688.2 | 1514.7 |
| 3 | 1913.6 | 1275.2 | 1507.5 | 1690.3 | 1506.2 |
| 4 | 1924.5 | 1284.6 | 1514.7 | 1691.2 | 1610.6 |
| 5 | 1929.6 | 1297.2 | 1512.1 | 1683.7 | 1616.0 |
| 6 | 1994.8 | 1296.6 | 1522.5 | 1689.4 | 1518.6 |
| 7 | 1918.6 | 1296.1 | 1503.2 | 1689.9 | 1413.3 |
| 8 | 1918.5 | 1288.7 | 1524.3 | 1689.0 | 1501.7 |
| 9 | 1921.8 | 1286.6 | 1510.4 | 1674.1 | 1508.6 |
| 10 | 1950.1 | 1286.3 | 1509.1 | 1686.8 | 1498.9 |
| 11 | 2005.9 | 1286.6 | 1527.4 | 1679.3 | 1514.7 |
| 12 | 1998.4 | 1293.8 | 1519.4 | 1675.2 | 1469.8 |
| 13 | 2015.6 | 1276.1 | 1517.5 | 1691.8 | 1498.3 |
| 14 | 2079.5 | 1276.6 | 1517.3 | 1682.6 | 1416.8 |
| 15 | 2142.2 | 1279.8 | 1528.1 | 1689.2 | 1492.2 |
| 16 | 2160.4 | 1285.6 | 1524.5 | 1691.7 | 1508.8 |
| 17 | 2084.5 | 1288.5 | 1521.2 | 1666.9 | 1508.5 |
| 18 | 2094.2 | 1287.6 | 1507.1 | 1690.3 | 1411.3 |
| 19 | 2125.8 | 1284.6 | 1524.3 | 1688.1 | 1400.1 |
| 20 | 2123.7 | 1284.5 | 1504.2 | 1689.6 | 1503.9 |
| 21 | 2011.2 | 1286.8 | 1515.0 | 1714.2 | 1631.8 |
| 22 | 2126.5 | 1287.6 | 1516.3 | 1690.2 | 1621.9 |
| 23 | 1967.5 | 1294.5 | 1519.2 | 1630.8 | 1637.7 |
| 24 | 1923.8 | 1287.7 | 1510.1 | 1689.3 | 1505.6 |
| 25 | 1932.5 | 1283.7 | 1515.1 | 1689.9 | 1512.9 |
| Average | 2003.6 | 1286.8 | 1515.8 | 1686.5 | 1510.0 |
| Standard deviation | 87.04 | 5.91 | 7.08 | 15.79 | 68.99 |

|  | Attenuation coefficient (dB/cm-MHz) | | | | |
| --- | --- | --- | --- | --- | --- |
|  | General-purpose  shrink tube | Silicone shrink  tube | Polyolefin shrink  tube | General- purpose  vinyl tube | Bovine vessel  tissue |
| 1 | 18.240 | 2.920 | 0.846 | 10.509 | 1.295 |
| 2 | 19.640 | 2.900 | 0.841 | 11.408 | 1.295 |
| 3 | 18.210 | 2.742 | 0.834 | 10.692 | 1.287 |
| 4 | 18.660 | 2.890 | 0.894 | 9.218 | 1.139 |
| 5 | 17.880 | 2.880 | 0.889 | 9.518 | 1.430 |
| 6 | 19.390 | 2.996 | 0.849 | 13.583 | 1.095 |
| 7 | 18.210 | 2.461 | 1.442 | 10.554 | 1.184 |
| 8 | 18.830 | 2.672 | 1.535 | 11.002 | 1.980 |
| 9 | 17.980 | 2.834 | 1.556 | 10.708 | 1.067 |
| 10 | 18.390 | 2.498 | 0.847 | 10.763 | 1.198 |
| 11 | 19.220 | 2.512 | 0.845 | 11.199 | 1.098 |
| 12 | 17.910 | 2.446 | 0.849 | 10.543 | 1.094 |
| 13 | 17.930 | 2.186 | 1.574 | 10.552 | 1.128 |
| 14 | 19.440 | 2.442 | 1.546 | 12.306 | 1.796 |
| 15 | 18.520 | 2.668 | 1.564 | 10.828 | 1.125 |
| 16 | 18.550 | 3.824 | 1.555 | 10.663 | 1.062 |
| 17 | 18.630 | 3.665 | 1.541 | 10.902 | 1.820 |
| 18 | 17.870 | 3.602 | 1.346 | 10.521 | 1.099 |
| 19 | 18.330 | 3.669 | 0.844 | 10.853 | 1.130 |
| 20 | 19.380 | 3.548 | 0.916 | 13.276 | 2.063 |
| 21 | 17.800 | 2.994 | 0.885 | 9.788 | 2.136 |
| 22 | 17.960 | 2.175 | 0.884 | 10.527 | 1.336 |
| 23 | 19.370 | 2.194 | 1.524 | 11.573 | 2.174 |
| 24 | 18.950 | 2.186 | 1.563 | 11.063 | 2.084 |
| 25 | 18.520 | 2.104 | 1.543 | 12.948 | 2.125 |
| Average | 18.55 | 2.800 | 1.180 | 11.02 | 1.45 |
| Standard deviation | 0.58 | 0.52 | 0.34 | 1.05 | 0.418 |

|  | Density (g/cm^3^) | | | | |
| --- | --- | --- | --- | --- | --- |
|  | General-purpose  shrink tube | Silicone shrink  tube | Polyolefin shrink  tube | General- purpose  vinyl tube | Bovine vessel  tissue |
| 1 | 1.1961 | 1.0807 | 0.9231 | 1.1728 | 1.0987 |
| 2 | 1.2016 | 1.0683 | 0.9433 | 1.1835 | 1.1184 |
| 3 | 1.2129 | 1.0351 | 0.8902 | 1.1655 | 1.1426 |
| 4 | 1.1930 | 1.1101 | 0.8939 | 1.1643 | 1.0332 |
| 5 | 1.1899 | 1.0977 | 0.8756 | 1.2098 | 1.0501 |
| 6 | 1.1500 | 1.1175 | 0.9189 | 1.1300 | 1.1669 |
| 7 | 1.2061 | 1.1095 | 0.9859 | 1.1610 | 1.2588 |
| 8 | 1.2124 | 1.1384 | 0.9171 | 1.1362 | 1.0528 |
| 9 | 1.1937 | 1.0804 | 0.9117 | 1.1941 | 1.1919 |
| 10 | 1.1887 | 1.1024 | 0.8959 | 1.1667 | 1.1128 |
| 11 | 1.1386 | 1.0944 | 0.9467 | 1.1326 | 1.0576 |
| 12 | 1.1569 | 1.1138 | 0.9096 | 1.1736 | 1.1355 |
| 13 | 1.1471 | 1.1535 | 0.9140 | 1.0882 | 1.0605 |
| 14 | 1.0796 | 1.1491 | 0.8799 | 1.1458 | 1.1181 |
| 15 | 1.0443 | 1.1010 | 0.9149 | 1.1716 | 1.0689 |
| 16 | 1.0517 | 1.0916 | 0.9151 | 1.1373 | 1.0585 |
| 17 | 1.1029 | 1.1000 | 0.9098 | 1.1884 | 1.1767 |
| 18 | 1.0381 | 1.0966 | 0.9230 | 1.1489 | 1.1812 |
| 19 | 1.0801 | 1.0961 | 0.8738 | 1.1777 | 1.1295 |
| 20 | 1.0585 | 1.1226 | 0.9693 | 1.0961 | 1.0626 |
| 21 | 1.1361 | 1.0981 | 0.9399 | 1.1883 | 0.9977 |
| 22 | 1.0886 | 1.0935 | 0.9174 | 1.1354 | 1.0605 |
| 23 | 1.1741 | 1.0807 | 0.9420 | 1.1730 | 1.0875 |
| 24 | 1.2039 | 1.1260 | 0.9132 | 1.1827 | 1.1517 |
| 25 | 1.1902 | 1.0937 | 0.9630 | 1.1492 | 1.1197 |
| Average | 1.145 | 1.102 | 0.919 | 1.159 | 1.108 |
| Standard deviation | 0.060 | 0.025 | 0.028 | 0.029 | 0.06 |

|  | Acoustic impedance (Mrayls) | | | | |
| --- | --- | --- | --- | --- | --- |
|  | General-purpose  shrink tube | Silicone shrink  tube | Polyolefin shrink  tube | General- purpose  vinyl tube | Bovine vessel  tissue |
| 1 | 2.290 | 1.392 | 1.394 | 2.018 | 1.568 |
| 2 | 2.298 | 1.374 | 1.429 | 1.998 | 1.694 |
| 3 | 2.321 | 1.320 | 1.342 | 1.970 | 1.721 |
| 4 | 2.296 | 1.426 | 1.354 | 1.969 | 1.664 |
| 5 | 2.296 | 1.424 | 1.324 | 2.037 | 1.697 |
| 6 | 2.294 | 1.449 | 1.399 | 1.909 | 1.772 |
| 7 | 2.314 | 1.438 | 1.482 | 1.962 | 1.779 |
| 8 | 2.326 | 1.467 | 1.398 | 1.919 | 1.581 |
| 9 | 2.294 | 1.390 | 1.377 | 1.999 | 1.798 |
| 10 | 2.318 | 1.418 | 1.352 | 1.968 | 1.668 |
| 11 | 2.284 | 1.408 | 1.446 | 1.902 | 1.602 |
| 12 | 2.312 | 1.441 | 1.382 | 1.966 | 1.669 |
| 13 | 2.312 | 1.472 | 1.387 | 1.841 | 1.589 |
| 14 | 2.245 | 1.467 | 1.335 | 1.928 | 1.584 |
| 15 | 2.237 | 1.409 | 1.398 | 1.979 | 1.595 |
| 16 | 2.272 | 1.403 | 1.395 | 1.924 | 1.597 |
| 17 | 2.299 | 1.417 | 1.384 | 1.981 | 1.775 |
| 18 | 2.174 | 1.412 | 1.391 | 1.942 | 1.667 |
| 19 | 2.296 | 1.408 | 1.332 | 1.988 | 1.581 |
| 20 | 2.248 | 1.442 | 1.458 | 1.852 | 1.598 |
| 21 | 2.285 | 1.413 | 1.424 | 2.037 | 1.628 |
| 22 | 2.315 | 1.408 | 1.391 | 1.919 | 1.720 |
| 23 | 2.310 | 1.399 | 1.431 | 1.913 | 1.781 |
| 24 | 2.316 | 1.450 | 1.379 | 1.998 | 1.734 |
| 25 | 2.300 | 1.404 | 1.459 | 1.942 | 1.694 |
| Average | 2.290 | 1.418 | 1.394 | 1.954 | 1.670 |
| Standard deviation | 0.034 | 0.033 | 0.042 | 0.050 | 0.08 |
